# The Body Knows What the Mind Does Not: Uncertainty Affects Physiological Markers of Deception

**DOI:** 10.1111/psyp.70273

**Published:** 2026-03-12

**Authors:** Giulia Romano Cappi, Ilaria Mirlisenna, Alessandro Mazza, Olga Dal Monte

**Affiliations:** ^1^ Department of Psychology University of Turin Italy; ^2^ Center for Mind/Brain Sciences (CIMeC), University of Trento Rovereto Italy; ^3^ NIT (Neuroscience Institute of Turin) Italy; ^4^ Department of Psychology Yale University New Haven Connecticut USA

**Keywords:** deception, electrodermal activity, facial electromyography, lying, self‐deception, self‐enhancement, uncertainty

## Abstract

Humans, as social beings, may choose to be either truthful or deceptive to achieve personal benefits. Although deception and truth‐telling are distinct, both rely on an individual's certainty about the world, shaping responses associated with honesty or lying. However, our surrounding environment is often characterized by a lack of certainty, and little is known about the psychophysiological responses in situations of uncertainty, where individuals lack confidence in whether their statements are truthful or deceptive. In this study, we implemented a within‐subject design where thirty‐two participants (mean age: 23.94; age range: 19–32; 17 females) were asked to persuade another person about the goodness of their performance in cognitively demanding tasks, while their self‐confidence, electrodermal activity (EDA), and facial muscle activity were measured under three conditions: Deception, Uncertainty, and Truth. Participants reported lower self‐confidence in convincing an interlocutor of their great results during Deception compared to Truth. Deceptive behavior was also associated with increased physiological arousal (trough‐to‐peak; TTP) compared to truth‐telling. Crucially, when faced with uncertainty, participants exhibited behavioral and physiological responses that fell in between those of Deception and Truth, self‐enhancing their performance while retaining residual characteristics of deception at the implicit level. We propose that these results could be framed within a continuum between truth and deception, where uncertainty can give rise to a form of partial deception, allowing individuals to enhance their self‐confidence while reducing the social and physiological drawbacks of lying.

## Impact Statement

Deception and truth‐telling rely on an individual being certain about the state of the world. While much research has been done on deceptive and truth‐telling behaviors, less is known about psychophysiological correlates of uncertainty, that is, when individuals cannot confidently know if they are lying or telling the truth, due to a lack of information. In this study, we examined for the first time both the explicit (self‐confidence) and implicit (electrodermal and facial muscle activity) responses to uncertainty. Our findings suggest that responses to uncertain stimuli tend to fall between those typically associated with deception and truth. These results support the idea of a continuum between truth and deception, where uncertainty can lead to a partial lie or self‐deception, enabling individuals to enhance their self‐confidence while minimizing the social and physiological costs of lying.

## 1 Introduction

As social creatures, humans are inherently driven by a deep‐seated desire to belong and be acknowledged as members of a social group. To achieve high‐status positions, individuals frequently engage in deceiving behaviors to reap the benefits associated with a higher social status, such as access to resources, increased reproductive success and survival, positing deception as an advantageous selective behavior (Anderson and Kilduff 2009; Bond and Robinson 1988; Butterworth et al. 2022). However, deception can also be associated with observable external cues or tell‐tale signs (Krstić 2019, 2020), such as sweating (Jiang 2022), blushing (Orne et al. 1972), fidgeting (DePaulo et al. 2003), and nervousness (Von Hippel and Trivers 2011), which can potentially lead to detection and ultimately expose the individual to the risk of social rejection (Trivers 2010).

It is indeed well known that lying is associated with rapid autonomic reactions, such as an increase in electrodermal activity (EDA) that reflects the implicit physiological counterpart of visible tell‐tale signs (Aranjo 2021; Jiang 2022; Pennebaker and Chew 1985; Pyasik and Pia 2021). In contrast, honesty is associated with lower EDA (Gödert et al. 2001; Ströfer et al. 2015) and appears to promote general arousal down‐regulation (ten Brinke et al. 2015). Facial muscles, such as Corrugator Supercilii and Zygomaticus Major, play an important role in deceptive behaviors as well (Dong et al. 2022). Indeed, individuals tend to inhibit facial expressions when lying to avoid lie detection and the resulting risk of social rejection (Ekman and Friesen 1969; Ekman and O'Sullivan 2006; Frank and Svetieva 2015; Shuster et al. 2021; ten Brinke et al. 2012). However, studies using facial electromyography (fEMG) to directly investigate facial muscle activity during deception and honesty have yielded conflicting results (Dong et al. 2025; ten Brinke et al. 2012), making further investigation essential to fill this gap.

A common feature of both deception and truth‐telling is that they both rely on individuals' certainty about the state of the world (Yang et al. 2019). When a fact is known, a person can choose to tell either a congruent or incongruent story with respect to the known fact, thus deliberately choosing to either be honest or lie, respectively. While behavioral and physiological manifestations of deception and truth‐telling have been extensively studied (de Vries et al. 2020; DePaulo et al. 2003; Jiang 2022; Littlefield et al. 2015), much less is known about how individuals navigate situations of uncertainty.

Imagine you are a student about to take an important exam. At the start of the term, you were told that only the top‐performing student in the class would be invited to attend a prestigious seminar offering the chance to meet an esteemed scientist. After completing the written exam, the teaching assistant briefly reviews your test. She/He provides vague feedback, telling you that you probably performed well but without offering a definitive score, as all the exams still need to be fully graded. Shortly after, your professor asks you to evaluate your performance. Naturally, you want to convince the professor of your excellent results, and you will likely emphasize how well you performed. This represents a situation in which individuals cannot confidently determine whether they are lying or telling the truth because they lack certain information about their performance. Thus, when individuals have only a vague knowledge of their own performance but need to persuade someone of their exceptional results, do they respond with honesty‐like behaviors, or do they exhibit tell‐tale signs of deception?

Theoretically, if we conceptualize a continuum between complete adherence to known, certain facts (i.e., Truth; de Vries et al. 2020; Littlefield et al. 2015), and intentional manipulation of information for personal profits (i.e., Deception; DePaulo et al. 2003; Jiang 2022), uncertainty occupies a middle ground, introducing a factor of ambiguity that may elicit unique behavioral and physiological responses. Additionally, it is well known that humans tend to show a positive bias towards an overestimation of their own qualities and attributes (Heine et al. 1999; Sedikides et al. 2003), to fulfill their functional need for social recognition and social belonging (Anderson et al. 2012). While it remains unclear whether these self‐enhancing tendencies arise in conditions of uncertainty, it may be possible that they allow individuals to fill the gaps in their knowledge when relying on limited or probabilistic information.

Here, we aimed to investigate the behavioral and physiological reactions when individuals find themselves in a situation of uncertainty. By adopting a social interactive paradigm, participants were instructed to persuade another individual of having performed among the best in a series of cognitive tasks, regardless of their actual performance. To achieve this goal, we manipulated the feedback they received from a computer about their performance, which could be either *Certain* and *Positive* (i.e., “You *certainly* performed among the best”; Truth conditions), *Certain* and *Negative* (i.e., “You *certainly* performed among the worst”; Deception conditions), or *Uncertain* (“You *probably* performed among the best/worst”; Uncertain conditions). By instructing participants to consistently state of performing well, our aim was to investigate how Deception, Uncertainty, and Truth conditions could influence their behavioral and physiological responses at the explicit (Self‐Confidence ratings) and implicit (EDA and fEMG) levels.

In line with previous literature (Ekman and Friesen 1969; Jiang 2022; Peifer et al. 2020), we first hypothesized that: (i) Deception would be associated with lower Self‐Confidence ratings compared to Truth; (ii) Deception would also elicit higher EDA than Truth, reflecting greater physiological arousal; (iii) Deception would be associated with a decrease in facial muscular activity compared to Truth, reflecting emotional inhibition. Crucially, our main goal was to explore explicit and implicit responses to Uncertainty by testing two alternatives: if uncertainty preferentially drives individuals to emphasize partial truths, then we should observe Truth‐like responses; conversely, if self‐enhancement works through deliberate deceptive exaggerations, then we should observe Deception‐like reactions. Specifically, since we speculate that self‐enhancement does not involve outright lies, yet a genuine overemphasis of positive self‐attributes, we expected to observe Truth‐like responses for both explicit and implicit measures.

## 2 Materials and Methods

### 2.1 Participants

Thirty‐two healthy volunteers (Biological sex: 17 females and 15 males; age range: 19–32; mean age = 23.94 ± 3), all residing in Italy and speaking the local language, took part in the study. Participants (undergraduate and graduate students) were recruited via social media platforms and snowball sampling. We verbally assessed that participants did not have any history of neurological or psychiatric disease and signed the written informed consent before taking part in this experiment. The study was not preregistered, but the experimental procedures were approved by the local committee (n. 0183596) and conducted in accordance with the Declaration of Helsinki (World Medical Association 2013). At the end of the experiment, all participants were informed about the aims and scopes of the experiment and did not receive any compensation for participation in this research study.

### 2.2 Experimental Setting, Task and Design

**FIGURE 1:**
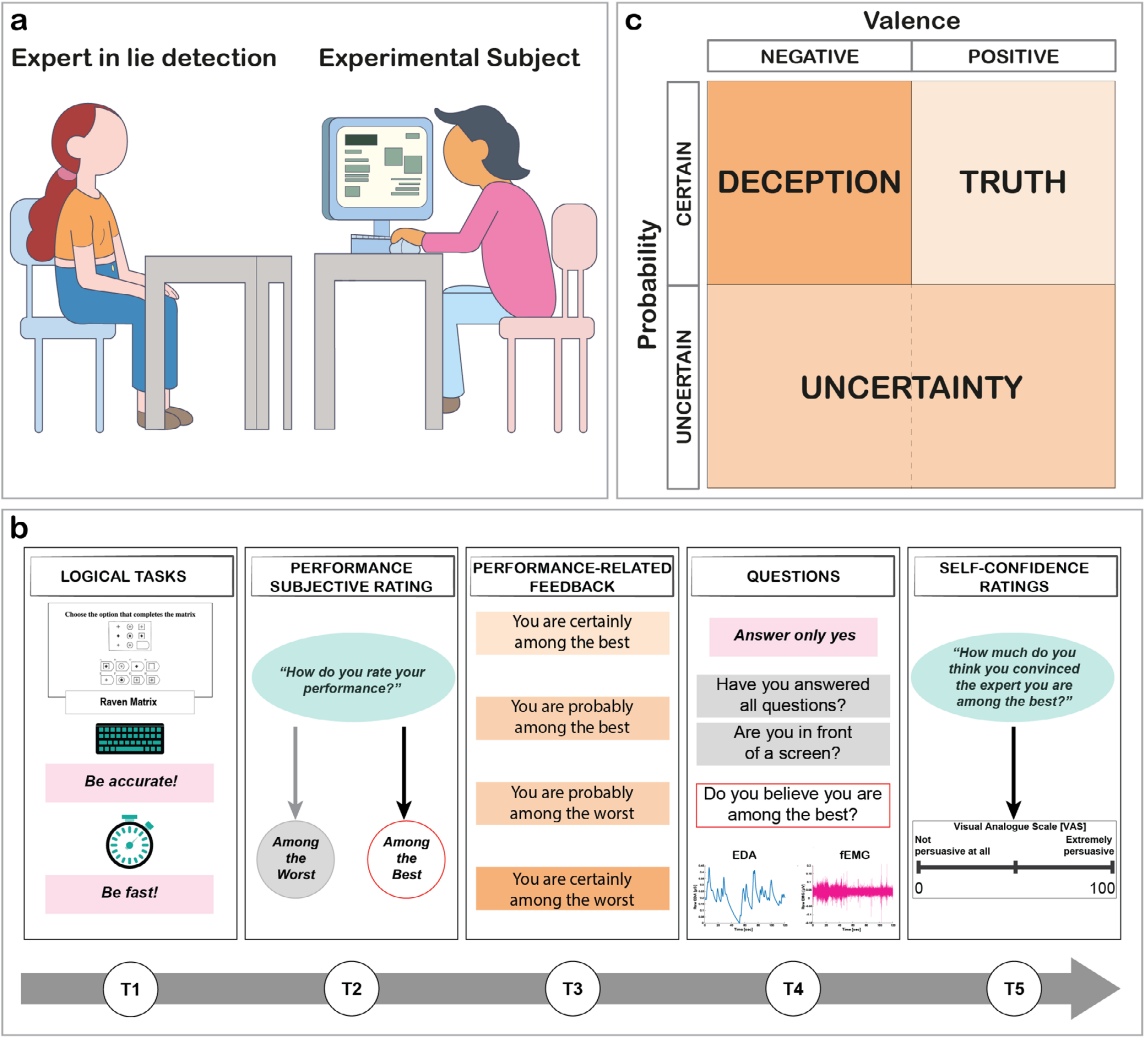
Setting, Design, and Experimental Conditions. (a) Experimental Setting: Participants (right) sat in front of a confederate (*expert in lie detection*, left). Participants used a computer for the whole duration of the experiment, apart from a few moments during which they interacted with the *expert in lie detection*. (b) Experimental Design: Each trial followed a consistent structure. First (T1), participants engaged in solving four distinct logical tasks (e.g., Raven Matrices and Proportions) presented as a multiple‐choice question. These tasks were displayed on a computer monitor and were completed using the computer keyboard. A count‐down was also displayed on the screen, allowing participants to monitor the time elapsed (with a maximum of 30 s allocated for each task). After completing the logical tasks, participants were required to provide subjective ratings of their performance (Performance Subjective Rating, T2). They used the left and right keys on the keyboard to rate their performance as negative or positive, respectively. Then, a Performance‐related Feedback (T3) was presented on the screen, displaying four possible outcomes: “You are certainly among the best” (*Certain Positive*), “You are probably among the best” (*Uncertain Positive*), “You are probably among the worst” (*Uncertain Negative*), and “You are certainly among the worst” (*Certain Negative*). Following the Performance‐related Feedback, participants were invited to engage in an interaction with the *expert in lie detection*. Their objective was to persuade the expert that they ranked among the best, regardless of their actual scores. They were given clear instructions to respond with only “yes” to the expert's questions, and their physiological data, including electrodermal activity and facial electromyography, were recorded during this phase (T4). Finally, using a Visual Analogue Scale that ranged from 0 (“Not persuasive at all”) to 100 (“Extremely Persuasive”), participants were asked to rate the extent to which they believed they had convinced the *expert in lie detection* that they performed Among the Best (“How much do you think you have convinced the expert you are among the best?”). This self‐assessment was referred to as Self‐Confidence ratings (T5). (**c**) Experimental conditions of Deception, Uncertainty, and Truth emerged from the Performance‐related Feedback. *Certain* and *Negative* Performance‐related Feedback defined Deception conditions. *Certain* and *Positive* Performance‐related Feedback defined Truth conditions. In contrast, *Uncertain* Performance‐related Feedback defined Uncertainty conditions, regardless of Valence.

The study employed a within‐subjects design, in which participants sat in front of a computer screen, while a female confederate sat behind the screen and played the role of *expert in lie detection* (Figure 1a). At the beginning of each trial (Figure 1b, **T1**), participants were invited to solve four different logical tasks. After completing the four tasks, participants were asked to rate the quality of their own performance by selecting one of two options displayed on the monitor: “I performed among the worst” or “I performed among the best” (Figure 1b, **T2**, Performance Subjective rating). Next, feedback about participants' performance was displayed on the screen, ranking them with respect to the other presumed participants. Participants could receive one out of four possible feedback, reflecting one of the four possible combinations of Probability (*Certain* vs. *Uncertain*) and Valence [*Positive* (i.e., Among the Best) vs. *Negative* (i.e., Among the Worst)] (Figure 1b, **T3**, Performance‐related Feedback). Importantly, at the end of each trial, participants were instructed to always convince the *expert in lie detection* that their performance was among the best. The type of Performance‐related Feedback they received defined the experimental conditions. Specifically, *Certain Positive* Performance‐related Feedback (“You are certainly among the best”) defined Truth conditions, as it aligned with the goal of convincing the *expert in lie detection* that they were among the best. Conversely, *Certain Negative* Performance‐related Feedback (“You are certainly among the worst”) defined Deception conditions, as it contradicted the goal of presenting themselves positively. Crucially, *Uncertain* Performance‐related Feedback [both *Positive* (“You are probably among the best”) and *Negative* (“You are probably among the worst”) Valence] defined Uncertainty conditions, as it left participants with vague and probabilistic knowledge about their actual performance, potentially encouraging them to further speculate about it to enhance their self‐confidence (Schwardmann and van der Weele 2019; Figure 1c). Importantly, the Performance‐related Feedback was randomly generated and did not reflect the actual performance of the subject. Next, participants were asked to interact with the *expert in lie detection* and to persuade her that they ranked among the best, regardless of the feedback they had just received. Specifically, participants were instructed to always answer “yes” to three questions posed by the *expert in lie detection*. The first two questions were control questions aimed at preventing possible startling responses occurring during the third experimental question (Pyasik and Pia 2021), while the third question actually probed the quality of the participants' performance (i.e., “Do you believe you are among the best?”). To motivate them to perform their best, participants were informed that they could earn points and receive a prize at the end of the session, based on their speed and accuracy relative to other presumed participants. Specifically, they were told that their chances of receiving the prize would depend on their accuracy and speed in solving tasks, as well as their ability to persuade the *expert in lie detection* of their strong performance (Figure 1b, **T4**). This approach was used because research has consistently shown that the potential for rewards enhances motivation and, in turn, increases engagement (David and Weinstein 2024; Ge et al. 2021; Ji et al. 2021; Lindström et al. 2021; Michaelsen and Esch 2021). Last, participants were asked to rate their confidence (Self‐Confidence ratings) in convincing the *expert in lie detection* by using a Visual Analogue Scale on the screen (i.e., “How much do you think you have convinced the expert you are among the best?”) ranging from 0 (“Not persuasive at all”) to 100 (“Extremely Persuasive”) (Figure 1b, **T5**). Experimental sessions consisted of 20 trials and lasted about 60 min. Physiological data (EDA and fEMG) were recorded throughout the whole experimental procedure.

### 2.3 Data Processing

#### 2.3.1 Self‐Confidence ratings

In each trial, Self‐Confidence ratings were quantified by recording participants' responses to the question “How much do you think you have convinced the expert you are among the best?”. Self‐Confidence ratings were calculated as the subjective scores (from 0 to 100) reported by the subjects for each trial.

#### 2.3.2 Physiological Measures

Before the beginning of the experimental session, electrodes for recording EDA and fEMG activity were placed on each participant. Physiological data were recorded using a BIOPAC MP160 (Biopac Systems Inc.) and visualized online using iMotions A/S Software 8.2. Physiological data were recorded through the whole experimental procedure, then the epochs of interest were extracted, consisting of the 11‐s window starting from the beginning of each question posed by the *expert in lie detection* (two control questions and third experimental question; see Experimental Setting, Task and Design) to the following 11 s. The 11‐s window consisted of one second of baseline, followed by 10 s of signal from which peak metrics were calculated.

#### 2.3.3 Electrodermal Activity

**FIGURE 2:**
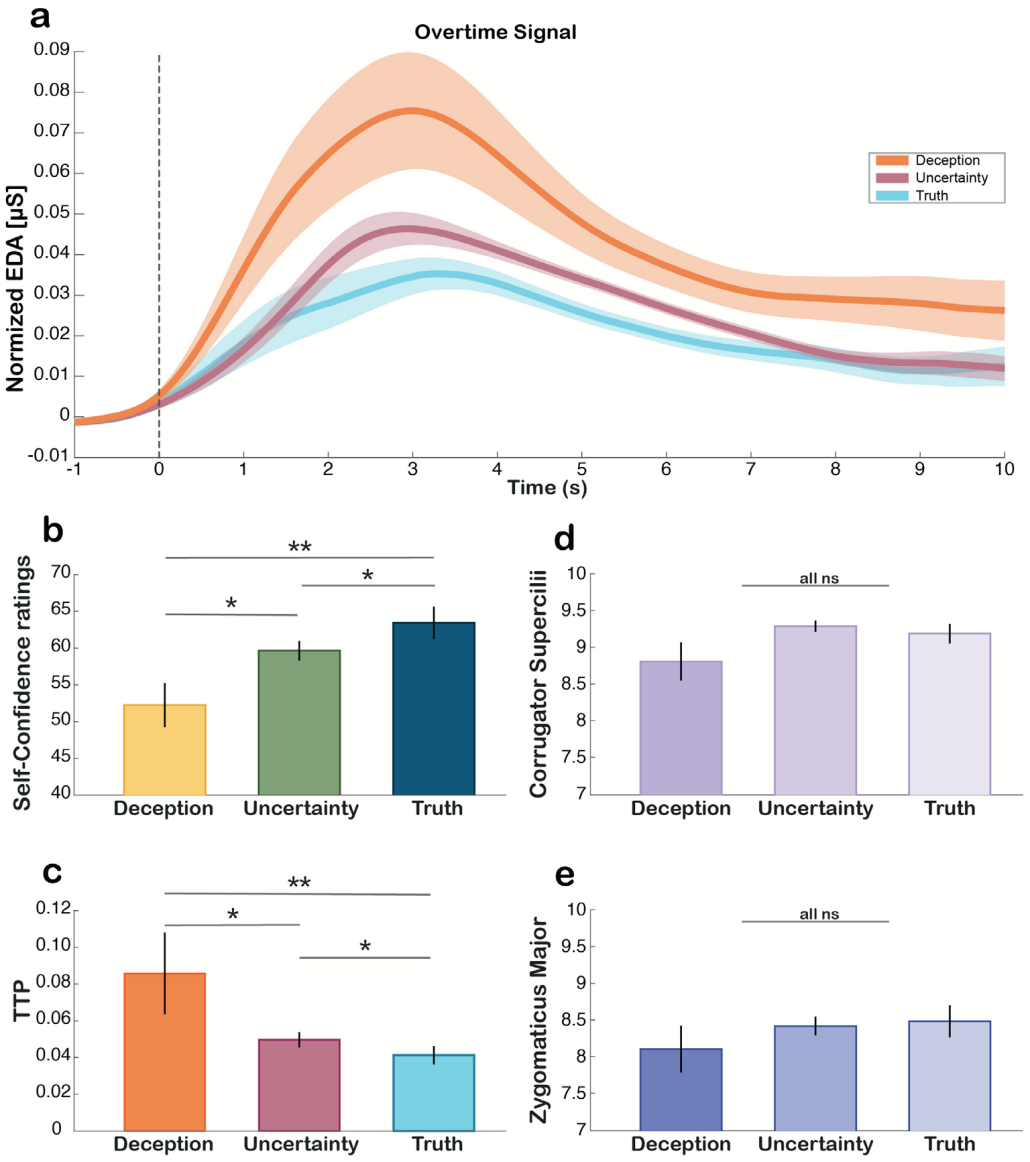
Behavioral and physiological responses. (a) Electrodermal Activity overtime for Deception, Uncertainty, and Truth conditions represented in orange, pink, and light blue, respectively. Time −1 represents the moment the expert in lie detection posited the experimental question; Time window [−1, 0] represents the baseline used for correcting the signal; Time window [0, 10] represents the signal from which trough‐to‐peak amplitude was calculated. Shaded traces represent ±0.5 standard error. (b) Self‐Confidence ratings for Deception, Uncertainty, and Truth conditions represented in yellow, green, and blue, respectively. Number of participants per condition: Deception = 27, Uncertainty = 32, Truth = 31. (c) Trough‐to‐peak amplitude for Deception, Uncertainty, and Truth conditions represented in orange, pink, and light blue, respectively. Number of participants per condition: Deception = 21, Uncertainty = 28, Truth = 29. (d) Corrugator Supercilii Duration for Deception, Uncertainty, and Truth conditions represented in progressively lighter shades of purple. Number of participants per condition: Deception = 26, Uncertainty = 31, Truth = 30. (e) Zygomaticus Major Duration for Deception, Uncertainty, and Truth conditions represented in progressively lighter shades of blue. Number of participants per condition: Deception = 27, Uncertainty = 32, Truth = 31. Bars represent the mean value for each measure and each condition. Error bars represent ±1 standard error. Asterisks indicate significant differences between Deception, Uncertainty, and Truth (* = *p* < 0.05; ** = *p* < 0.01).

EDA was recorded using the BIOPAC EDA100C module, applying two non‐polarizable Ag‐AgCl electrodes filled with GEL101 isotonic gel and attached to participants' proximal phalanges of the index and ring fingers. Data were acquired at a 500 Hz sampling rate, with a 2 μSiemens/Volt gain, and then processed offline with MATLAB r2024b. Specifically, for each trial, we extracted an 11‐s window of the signal starting from the onset of the question posited by the *expert in lie detection* (Figure 2a). The signal was first filtered with a 0.005 Hz high pass filter and a 1.0 Hz low pass filter in the whole‐trial period. The low pass filter was applied to remove noisy high‐frequency fluctuations, and the high pass filter ensured removing very slow tonic fluctuations. Then, in each 11‐s segment, the signal was further linearly detrended to exclude any residual tonic component in the epoch of interest. Finally, the first second was used to baseline‐correct the whole signal epoch, thus allowing to obtain a baseline‐corrected 10‐s signal following the question. The signal was then linearly transformed by subtracting the minimum value within the 10‐s window to rescale it around zero and facilitate visual inspection. Last, to filter out any residual high‐frequency fluctuation and avoid detection of spurious local maxima, the signal was smoothed using a moving average (*smoothdata* function, smoothing factor = 0.1). We then used the *findpeaks* function to identify local maxima and local minima of the signal in order to detect putative peaks and troughs, respectively. For each local maximum (peak) detected, the first local minimum preceding it was identified to localize the corresponding response onset (trough). Where more than one local minima and maxima were detected, we extracted the first trough‐peak pair satisfying the following criteria: a trough was considered valid if occurring between 1 to 3 s from time 0, and a peak was considered valid if occurring between 1 to 3 s after the trough (Dawson et al. 2007). When these conditions were met, trough‐to‐peak (TTP) amplitude was calculated as the difference between the peak amplitude and its corresponding trough. Trials where TTPs were lower than 0.01 microSiemens were not considered as a discrete response (Boucsein 2012) and excluded from subsequent analyses.

#### 2.3.4 Facial Electromyography

Corrugator Supercilii and Zygomaticus Major activities were recorded using two BIOPAC EMG100C modules. Before applying the electrodes, subjects' cheeks and foreheads were cleaned and exfoliated. For the corrugator, two Ag‐AgCl 4 mm electrodes were placed above the left side eyebrow. For the zygomaticus, two Ag‐AgCl 4 mm electrodes were placed on the left side cheek. Both muscles were recorded through bipolar electrodes positioned parallel to the muscle fiber direction (Lapatki et al. 2010). An Ag‐AgCl 4 mm electrode was used as ground and placed between the eyebrows. Importantly, electrodes position was adjusted based on each subject's anatomy. Specifically, each participant was asked to smile in order to identify the exact location of their Zygomaticus Major, and to frown to identify the exact location of their Corrugator Supercilii. Data were acquired at 500 Hz sampling rate, with a 2000× gain and 1.0 Hz online high pass filter. Data were then processed offline with MATLAB r2024b. First, signals were filtered with a 15 Hz high pass filter and a 50 Hz (and harmonics) notch filter, as recommended and implemented in previous studies (Boyer et al. 2023; Dong et al. 2025; Li et al. 2011). Then, for each trial, the absolute value of the signal and its envelope (Dong et al. 2025) were extracted. The 45% of the mean enveloped absolute signal was taken as threshold for subsequent processing (Özgünen et al. 2010). Finally, in order to extract fEMG duration, the amount of time during which the absolute enveloped signal was above threshold was calculated within the window of interest (i.e., the 10 s following the first second after the question). One subject was excluded from Corrugator Supercilii analyses due to problems during data collection.

### 2.4 Statistical Analyses

Since participants were asked to convince the *expert in lie detection* of the goodness of their performance, and our aim was to also investigate self‐enhancing behaviors (Dufner et al. 2019; Greenwald 1980; Gur and Sackeim 1979; Trivers 2000), only trials in which participants rated their performance positively (i.e., “I performed among the best”) were kept for statistical analyses (Heger and Papageorge 2018). Indeed, participants who rated their performance negatively are unlikely to display self‐enhancing behaviors—rather, they might go through a negative confirmation regarding their own performance or display surprised reactions to positive feedback. Next, for both behavioral (Self‐Confidence ratings) and physiological (TTP and fEMG duration) data, outlier trials were winsorized, that is, those falling above or under the 95th and 1st percentile of the respective measure were collapsed to the 95th and 1st percentile, respectively (Supporting Information). Winsorization is preferred over other methods when distributions are skewed (Abuzaid and Alkronz 2024), as in our case. Also, due to our choice to only retain trials in which participants rated their performance positively, we aimed at preserving as many observations as possible, and winsorization is preferred in doing so with respect to omission‐based methods involving outliers exclusion (Onoz and Oguz 2003). All subsequent analyses were run on MATLAB r2024b. Self‐Confidence ratings were analyzed employing a Linear Mixed‐Effects Model (LME). Since TTP and fEMG data showed skewed distributions, Generalized Linear Mixed‐Effects Models (GLMEs) for Gamma distributions with log link function were employed (Supporting Information). Since the two uncertain conditions had a different valence, we first ran a control analysis to explore possible differences between physiological responses to the two conditions (Supporting Information). Having confirmed that the two conditions were statistically comparable, we computed multiple models with measure of interest (Self‐Confidence ratings, TTP, fEMG duration) as dependent variable, Condition (Deception, Uncertainty, Truth) as single fixed‐effect predictor, and Subject as random effect. Lastly, post hoc pairwise comparisons were run by means of F‐test (MATLAB function *coefTest*) and corrected by means of false discovery rate (FDR; Benjamini and Hochberg 1995). All *p* values below 0.05 were interpreted as statistically significant for rejecting the null hypothesis.

## 3 Results

First, we computed a LME with Self‐Confidence ratings as the dependent variable, single fixed‐effect predictor Condition (Deception, Uncertainty, Truth), and Subject as a random effect. The model showed a significant effect of Condition [*F*_(2,376)_ = 5.845, *p* = 0.003, *η*_p_^2^ = 0.160; Figure 2b]. Post hoc pairwise comparisons showed that Deception was associated with lower Self‐Confidence ratings than Truth [*β* = −10.516, *t*_(141)_ = −3.375, *p* = 0.002], and Uncertainty [*β* = −6.026, *t*_(283)_ = −2.171, *p* = 0.033]. Moreover, Uncertainty was in turn associated with lower Self‐Confidence ratings than Truth [*β* = −4.491, *t*_(331)_ = −2.135, *p* = 0.033].

Next, we ran a GLME with TTP values as dependent variable, single fixed‐effect predictor Condition (Deception, Uncertainty, Truth), and Subject as random effect. We found that participants displayed distinct TTPs across Conditions [*F*_(2,258)_ = 6.440, *p* = 0.002, *η*_p_^2^ = 0.197; Figure 2c]. Post hoc pairwise comparisons showed that Deception was associated with a higher TTP amplitude compared to both Uncertainty [*β* = 0.213, *t*_(194)_ = 2.351, *p* = 0.029] and Truth [*β* = 0.365, *t*_(102)_ = 3.572, *p* = 0.001], while Uncertainty was associated with higher TTP than Truth [*β* = 0.152, *t*_(223)_ = 2.101, *p* = 0.037].

Lastly, we investigated whether the differences observed at the behavioral and electrodermal activity levels were reflected on facial muscle activity (fEMG). We computed a GLME with Corrugator Supercilii Duration values as dependent variable, single fixed‐effect predictor Condition (Deception, Uncertainty, Truth), and Subject as random effect. However, while the model was statistically significant [*F*_(2,354)_ = 3.036, *p* = 0.049, *η*_p_^2^ = 0.105; Figure 2d], no post hoc pairwise comparison was significant after FDR correction (all ps > 0.05). We additionally computed a GLME with Zygomaticus Major Duration values as dependent variable, single fixed‐effect predictor Condition (Deception, Uncertainty, Truth), and Subject as random effect, but no significant effect emerged from the model [*F*_(2,360)_ = 0.087, *p* = 0.916; Figure 2e].

## 4 Discussion

The present work investigated the behavioral and physiological responses when individuals hold only vague knowledge about the output of their own performance, but they are prompted to persuade another person of their exceptional achievement. As expected, participants reported lower self‐confidence in convincing an interlocutor of their great results during Deception compared to Truth conditions. Consistent with our hypotheses, we also observed that engaging in deceptive behaviors was linked to increased electrodermal activity. However, no significant difference was observed regarding muscle activity during deceptive or honest behavior. Crucially, uncertainty about the performance was associated with behavioral and physiological responses falling in between those of Deception and Truth.

On the explicit level, participants reported higher Self‐Confidence ratings when their performance was externally rated as excellent, and lower Self‐Confidence ratings when their performance was judged as poor. Notably, when they received uncertain feedback, participants reported Self‐Confidence ratings falling between those associated with Deception and Truth conditions. This suggests that participants may have self‐enhanced their perceived performance, potentially overestimating their abilities to resolve the uncertainty of the feedback, but not convinced themselves enough for their ratings to be comparable to those associated with excellent external ratings. On the implicit level, we found Deception to be associated with higher electrodermal responses compared to Truth. These results are consistent with previous literature and support the idea that deceptive behaviors are associated with increases in EDA (Ambach and Gamer 2018; Furedy and Ben‐Shakhar 1991; Lykken 1959, 1960; MacLaren 2001; Pennebaker and Chew 1985). However, while we expected to observe inhibition of facial expressions to facilitate the avoidance of lie detection (Ekman and Friesen 1969; Ekman and O'Sullivan 2006; Shuster et al. 2021; ten Brinke et al. 2012), no effect of our manipulation emerged on muscle activity. Indeed, previous studies reported decreased Corrugator Supercilii Activity associated with concomitant increases in EDA (Thompson et al. 2016), as well as larger Zygomaticus Major Activity during deceptive behaviors compared to truth‐telling (Dong et al. 2025; ten Brinke et al. 2012). Nonetheless, we did not observe significant differences across the experimental conditions for either the Corrugator Supercilii or the Zygomaticus Major Activity. Regarding the latter, since the Zygomaticus Major is typically associated with the expression of positive emotions (Dimberg et al. 1998; Dimberg and Thunberg 1998; Lindsey et al. 2011; Vrana and Gross 2004), we speculate that the lack of such preferential activation in our study could be due to the fact that neither deceptive nor truth‐telling behavior in this paradigm were specifically characterized by positive valence. Crucially, as observed for Self‐Confidence ratings, Uncertainty condition elicited electrodermal responses that sat between those of Deception and Truth. This suggests that while participants appeared to self‐enhance their perceived performance, residual characteristics associated with Deception persisted in Uncertainty condition.

We speculate that these results might be interpreted in a twofold way. First, we can consider Deception and Truth not as a binary concept, but as two extremes of a continuum within which the uncertain feedback could promote a “partial lie”. As such, participants might have been in a state where they were unable to be either fully deceptive or fully honest. In this case, self‐enhancing propensity could have boosted their confidence levels, allowing them to convince themselves of their good performance even in the absence of certain feedback. Nonetheless, this partial lie still retained deception‐like features that emerged at the electrodermal as well as the behavioral level. Second, the uncertainty of the feedback may have promoted self‐deceiving tendencies in participants. Indeed, self‐deception has been described as a “gray area” between deception and truth (Bicchieri et al. 2019) that emerges from uncertainty (Schwardmann and van der Weele 2019; Sloman et al. 2010) and allows individuals to self‐enhance themselves (Dufner et al. 2019; Trivers 2000), reflecting a natural mechanism capable of preserving the adaptive benefits of deception while counteracting its behavioral and physiological costs (Chance and Norton 2015; Smith et al. 2017; Trivers 2000). In this case, participants would not have been fully aware of their partial lie, thus deceiving themselves before deceiving another person. Crucially, EDA responses related to uncertain feedback would be coherent with the hypothesis that self‐deception exists to bolster individuals' self‐confidence while attenuating its physiological and behavioral manifestations (Trivers 2000, 2010; Von Hippel and Trivers 2011). Lastly, our results may be additionally explained based on the criteria that Gur and Sackeim (1979) presented for ascribing self‐deception. According to the authors, self‐deception entails holding two contradictory and concurrent beliefs, represented in this study as the positive Performance Subjective Rating and the *Uncertain* Performance‐related Feedback. Additionally, the individual must not be aware of holding one of these two beliefs, represented in this study as the preference participants displayed to keep their belief of having performed well, while ignoring the uncertain feedback. Lastly, the individual must be motivated to hold only one of the two beliefs, represented in this study as the final prize that participants concurred on. Considering these observations, our results support the idea that in situations where information is lacking, individuals tend to fill the gaps with positive false beliefs about themselves, deceiving themselves about their qualities before deceiving another person. However, at the same time, traces of the deceptive nature of the process continue to exist on an implicit level, as indicated by electrodermal activity results, as well as on an explicit level, as indicated by behavioral results. In this light, our findings represent a preliminary step towards understanding how self‐deceptive mechanisms may emerge under conditions of uncertainty, and how such processes may manifest at the physiological level. Future studies should aim to empirically validate this link by developing targeted paradigms capable of isolating and capturing the distinctive features of self‐deception at the implicit level.

Before concluding, we must acknowledge some limitations of the study, as well as additional possible directions for future research. First, considering the complexity of the experimental design of the present study, the sample size is relatively small, as reflected by the modest effect sizes reported in the results. Future studies would benefit from replicating these findings with larger samples to confirm their reliability and generalizability. Moreover, due to the experimental design and the theoretical rationale for retaining only trials in which participants rated their performance as among the best, the resulting number of trials per condition and measure was reduced and unbalanced. This imbalance may have affected the results by reducing comparability across conditions. Additionally, we recruited a homogeneous sample of subjects, specifically graduate and undergraduate Italian students. In the future, it would be valuable to explore more diverse populations, considering factors like age, nationality, and education to investigate potential individual differences. Additionally, although participants were fully debriefed about the aims and scopes of the study at the end of the experimental session, we only probed the positive outcome of the deceptive intentions (i.e., whether participants were indeed deceived) by collecting oral feedback. Future studies may benefit from employing standardized measures to quantify the degree of deception perceived by the participants throughout the experiment. Lastly, future studies should investigate the role of false feedback in subjects' experimental experience. While we are aware that false feedback is widely implemented in the neurophysiological field (Babür et al. 2024; Gino et al. 2010; Herzog and Fahle 1997; Shibata et al. 2009; Vuvan et al. 2018), we are also aware that it adds possible confounding variables to be taken into account. Along the same line, the promise of a reward to boost individuals' motivation and engagement should be more carefully controlled. While this expedient is widely used in the neurophysiological field as well (Clark et al. 2020; Jimura et al. 2010; Lindström et al. 2021), false feedback could potentially impact both ecological validity and general participants' trust towards researchers.

## 5 Conclusion

This study sheds light on the interplay between deception, uncertainty, and truth‐telling, offering valuable insights into how individuals navigate situations where information is partial and open to interpretation. Our findings show that uncertainty prompts self‐enhancing tendencies, allowing individuals to resolve ambiguity by overestimating their performance while residual characteristics of deception persist. These results are reflected in electrodermal and behavioral responses that fall in‐between those associated with deception and truth‐telling. These findings support the notion of a continuum between truth and deception, where uncertainty can foster a partial lie or self‐deception, enabling individuals to bolster their confidence while minimizing the social and physiological costs of lying. In summary, our study carries substantial implications for experimental and theoretical domains, offering new empirical insights into a mechanism that exists in the realm between deception and truth.

## Author Contributions

**Giulia Romano Cappi:** conceptualization, data curation, formal analysis, investigation, methodology, writing – review and editing, writing – original draft, visualization, validation, software. **Ilaria Mirlisenna:** conceptualization, data curation, formal analysis, investigation, software, validation, writing – original draft, writing – review and editing. **Alessandro Mazza:** conceptualization, methodology, software, validation, writing – review and editing. **Olga Dal Monte:** conceptualization, methodology, writing – review and editing, writing – original draft, resources, project administration, visualization, funding acquisition, formal analysis, supervision, validation, investigation, software, data curation.

## Conflicts of Interest

The authors declare no conflicts of interest.

## Supporting information


**Figure S1:** Behavioral and physiological data distributions. (a) Self‐Confidence ratings were modeled through a Linear Mixed‐Effects model. (b) Trough‐to‐peak values were modeled through a Generalized Linear Mixed‐Effects model with Gamma distribution and log link function. (c) and (d) fEMG duration values were first transformed (i.e., [$\left(x\times -1\right)+10$]), then modeled through a Generalized Linear Mixed‐Effects model with Gamma distribution and log link function.
**Figure S2:** Control Analyses. (a) Trough‐to‐peak amplitude for Negative Uncertainty and Positive Uncertainty conditions represented in light and dark pink, respectively. Since they were not significantly different, they were merged in a single Uncertainty condition. (b) Trough‐to‐peak amplitude (Question 1) for Deception, Uncertainty, and Truth conditions represented in orange, pink, and light blue, respectively. Number of participants per condition: Deception = 24, Uncertainty = 31, Truth = 24. (c) Trough‐to‐peak amplitude (Question 2) for Deception, Uncertainty, and Truth conditions represented in orange, pink, and light blue, respectively. Number of participants per condition: Deception = 17, Uncertainty = 27, Truth = 24. Bars represent the mean value for each condition. Error bars represent ±1 standard error. All comparisons are non‐significant (ns).
**Table S1:** Summary Table reporting Fixed Effects and Random Effects components.

## Data Availability

The data that support the findings of this study are openly available at https://github.com/SocialInteractionLabUnito/Physiological_Markers_Uncertainty.
