# Supplementary material for: The Body Knows What the Mind Does Not: Uncertainty Affects Physiological Markers of Deception
Source: Psychophysiology. 2026 Mar 12;63(3):e70273. doi: 10.1111/psyp.70273 (PMC12982658; doi:10.1111/psyp.70273)
Supplement: Supplementary file 1 — Figure S1: Behavioral and physiological data distributions. (a) Self‐Confidence ratings were modeled through a Linear Mixed‐Effects model. (b) Trough‐to‐peak values were modeled through a Generalized Linear Mixed‐Effects model with Gamma distribution and log link function. (c) and (d) fEMG duration values were first transformed (i.e., [x×−1+10]), then modeled through a Generalized Linear Mixed‐Effects model with Gamma distribution and log link function. Figure S2: Control Analyses. (a) Trough‐to‐peak amplitude for Negative Uncertainty and Positive Uncertainty conditions represented in light and dark pink, respectively. Since they were not significantly different, they were merged in a single Uncertainty condition. (b) Trough‐to‐peak amplitude (Question 1) for Deception, Uncertainty, and Truth conditions represented in orange, pink, and light blue, respectively. Number of participants per condition: Deception = 24, Uncertainty = 31, Truth = 24. (c) Trough‐to‐peak amplitude (Question 2) for Deception, Uncertainty, and Truth conditions represented in orange, pink, and light blue, respectively. Number of participants per condition: Deception = 17, Uncertainty = 27, Truth = 24. Bars represent the mean value for each condition. Error bars represent ±1 standard error. All comparisons are non‐significant (ns). Table S1: Summary Table reporting Fixed Effects and Random Effects components. [file PSYP-63-e70273-s001.docx]

**SUPPLEMENTARY INFORMATION**

Title

**The body knows what the mind does not: uncertainty affects physiological markers of deception**

Authors

Giulia Romano Cappi^1^, Ilaria Mirlisenna^1,2^, Alessandro Mazza^1^, Olga Dal Monte^1,3,4^

Affiliations

^1^ Department of Psychology, University of Turin, Torino, Italy

^2^ Center for Mind/Brain Sciences (CIMeC), University of Trento, Rovereto (TN), Italy

^3^ NIT (Neuroscience Institute of Turin) Turin, Italy

^4^ Department of Psychology, Yale University, New Haven, CT, USA

Authors email addresses:

Giulia Romano Cappi – [giulia.romanocappi@unito.it](mailto:giulia.romanocappi@unito.it)

Ilaria Mirlisenna – [ilaria.mirlisenna@unitn.it](mailto:ilaria.mirlisenna@unitn.it)

Alessandro Mazza – [alessandro.mazza@unito.it](mailto:alessandro.mazza@unito.it)

Olga Dal Monte – [olga.dalmonte@unito.it](mailto:olga.dalmonte@unito.it)

Corresponding Author:

Olga Dal Monte, Ph.D.

Via Verdi 10, 10124, Turin, Italy.

Email: [olga.dalmonte@unito.it](file:///C:\Users\francescacapiotto\Desktop\olga.dalmonte@unito.it)

Number of Figures: 2
Number of Tables: 1

**MATERIALS AND METHODS *Statistical Analyses***

Included and excluded trials

For the present study, only trials in which participants rated their performance as being among their best (Performance Subjective Rating) were retained for statistical analyses. On average, 61% of all recorded trials met this criterion and were included in all subsequent analyses, with the exception of the trough-to-peak (TTP) analyses. For TTP analyses, we applied an additional criterion, restricting the dataset to trials with TTP values greater than 0.01. This threshold resulted in the inclusion of 70% of the trials.

*Outlier cases*

Outlier cases falling below the 1^st^ percentile and above the 95^th^ percentile were winsorized. Specifically, for Self-Confidence Ratings, 4.2% of trials from *Deception*, 5.9% of trials from *Uncertainty*, and 6.3% of trials from *Truth* were winsorized. For TTP, 5.4% of trials from *Deception*, 6.3% of trials from *Uncertainty*, and 6.1% of trials from *Truth* were winsorized. For Corrugator Supercilii Duration, 4.4% of trials from *Deception*, 4.9% of trials from *Uncertainty*, and 4.5% of trials from *Truth* were winsorized. For Zygomaticus Major Duration, 4.3% of trials from *Deception*, 4.9% of trials from *Uncertainty*, and 5.5% of trials from *Truth* were winsorized.

Models

*Self-Confidence ratings*

Since data from Self-Confidence ratings showed a normal distribution (**Fig. S1a)**, two Linear Mixed-Effects Models (LMEs) were computed:

- Self-Confidence ratings (Uncertainty) ~ Valence + (1|Subject)

Where *Self-Confidence rating (Uncertainty)* was the dependent variable, with values from trials characterized by Uncertainty conditions, *Valence* (Positive, Negative) was the fixed effect, *Subject* was the random effect.

- Self-Confidence ratings ~ Conditions + (1|Subject)

Where *Self-Confidence rating* was the dependent variable, with values from all trials, *Conditions* (Deception, Uncertainty, Truth) was the fixed effect, *Subject* was the random effect.

*Trough-to-peak*

Since data from TTP showed a gamma distribution (**Fig. S1b)**, two Generalized Linear Mixed-Effects Models (GLMEs) were computed. The distribution was defined as “Gamma” and the Link Function was defined as “Log”:

- TTP (Uncertainty) ~ Valence + (1|Subject)

Where *TTP (Uncertainty)* was the dependent variable, with values from trials characterized by Uncertainty conditions, *Valence* (Positive, Negative) was the fixed effect, *Subject* was the random effect.

- TTP ~ Conditions + (1|Subject)

Where *TTP* was the dependent variable, with values from all trials, *Conditions* (Deception, Uncertainty, Truth) was the fixed effect, *Subject* was the random effect.


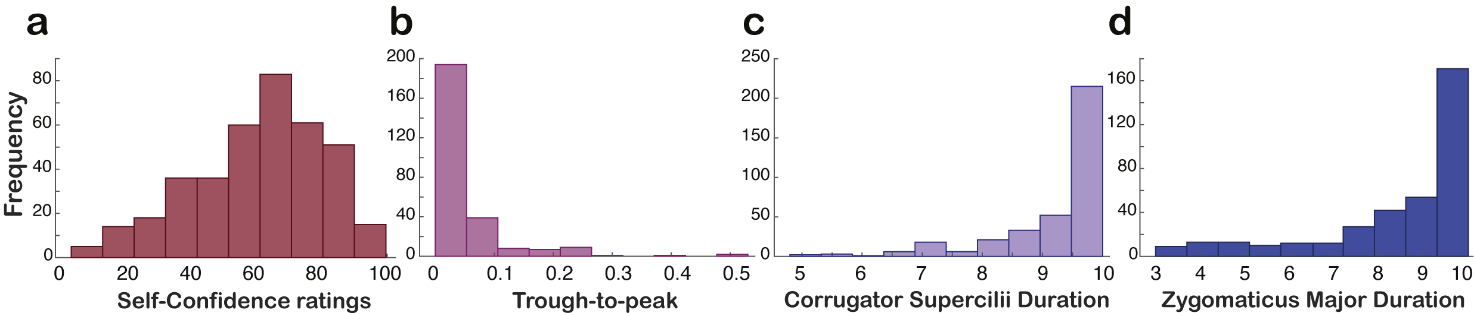


**Figure S1: Behavioral and physiological data distributions. a) Self-Confidence ratings** were modeled through a Linear Mixed-Effects model. **b) Trough-to-peak values** were modeled through a Generalized Linear Mixed-Effects model with Gamma distribution and log link function. **c) and d) fEMG duration values** were first transformed (i.e., [$\left( x\times-1 \right)+10$]), then modeled through a Generalized Linear Mixed-Effects model with Gamma distribution and log link function.

*Corrugator Supercilii Duration*

Since data from Corrugator Supercilii Duration showed an inversed gamma distribution (**Fig. S1c)**, two Generalized Linear Mixed-Effects Models (GLMEs) were computed, after transforming values so that they would distribute as a gamma distribution. The distribution was defined as “Gamma” and the Link Function was defined as “Log”:

- Corrugator Supercilii Duration (Uncertainty) ~ Valence + (1|Subject)

Where *Corrugator Supercilii Duration (Uncertainty)* was the dependent variable, with values from trials characterized by Uncertainty conditions, *Valence* (Positive, Negative) was the fixed effect, *Subject* was the random effect.

- Corrugator Supercilii Duration ~ Valence*Probability + (1|Subject)

Where *Corrugator Supercilii Duration* was the dependent variable, with values from all trials, *Valence* (Positive, Negative) and *Probability* (Certain, Uncertain) were the fixed effects, *Subject* was the random effect.

*Zygomaticus Major Duration*

Since data from Zygomaticus Major Duration showed an inversed gamma distribution (**Fig. S1d)**, two Generalized Linear Mixed-Effects Models (GLMEs) were computed, after transforming values so that they would distribute as a gamma distribution. The distribution was defined as “Gamma” and the Link Function was defined as “Log”:

- Zygomaticus Major Duration (Uncertainty) ~ Valence + (1|Subject)

Where *Zygomaticus Major Duration (Uncertainty)* was the dependent variable, with values from trials characterized by Uncertainty conditions, *Valence* (Positive, Negative) was the fixed effect, *Subject* was the random effect.

- Zygomaticus Major Duration ~ Valence*Probability + (1|Subject)

Where *Zygomaticus Major Duration* was the dependent variable, with values from all trials, *Valence* (Positive, Negative) and *Probability* (Certain, Uncertain) were the fixed effects, *Subject* was the random effect.

For all models, post-hoc pairwise comparisons were computed using the MATLAB function *coefTest*. Partial eta squared was computed as follows:

1. R^2^ was calculated from the full model
2. Pseudo R^2^ was calculated from the reduced model (i.e., model without the fixed effects)
3. Cohen’s *f* was calculated as:

$$f=\sqrt{\frac{R_{Full}^{2} - R_{Pseudo}^{2}}{1 - R_{Full}^{2}}}$$

4. Partial Eta squared $\eta_{p}^{2}$ was calculated as: $\frac{f}{1+f}$

Importantly, partial eta squared for the GLME with Zygomaticus Major Duration values reported in the main text could not be reported because the R^2^ calculated from the full model was smaller than the R^2^ calculated from the pseudo model.

***Control Analyses***

*Uncertainty Conditions*

Given that for the main analyses Uncertainty conditions were defined by the Probability of the Performance-related Feedback, irrespective of the Valence, two distinct Uncertainty conditions could stem from both the Uncertain Positive (i.e., “You are probably among the best”) and Uncertain Negative (i.e., “You are probably among the worst”) Performance-related Feedback. As such, control analyses were conducted to test whether positive and negative feedback were statistically different for TTP. TTP data showed skewed distributions, thus, a Generalized Linear Mixed-Effects Model (GLME) for Gamma distributions with log link function was computed. TTP values from trials where the Performance-related Feedback was either Uncertain Negative or Uncertain Positive were the dependent variable, Valence was the fixed-effect, and Subject was the random effect. Since the two conditions were statistically comparable, for all subsequent analyses, models were computed with single fixed-effect predictor Condition (Deception, Uncertainty, Truth).

*Control Questions*

To ensure that results on participants’ responses during the experimental question were ascribable to our experimental manipulation rather than to confounding variables, we replicated the statistical analyses on the two control questions. Notably, subjects’ physiological activation was not expected to be influenced by Condition (Deception, Uncertainty, and Truth) during either the first or the second control questions.

**RESULTS**

***Models: Fixed and Random Effects components***

**Table S1: Summary Table** reporting Fixed Effects and Random Effects components

***Uncertainty conditions***

We computed a GLME with TTP values from trials where the Performance-related Feedback was either Uncertain Negative or Uncertain Positive as dependent variable, Valence as fixed-effect, and Subject as random effect and found no significant difference between the two conditions [β = 0.015, t_(156)_ = 0.174, *p* = 0.862; **Fig. S2a**]. As such, we decided to merge the two uncertain conditions and run a GLME with single fixed-effect predictor Condition (Deception, Uncertainty, Truth).

***Control Analyses for Control Questions***

The GLME on TTPs did not show any significant effect of Conditions for either the first [F_(2, 222)_ = 1.118, p = 0.329, η_p_^2^ = 0.073; **Fig. S2b**] or the second [F_(2, 210)_ = 0.201, p = 0.818, η_p_^2^ = 0.055; **Fig. S2c**] control questions.


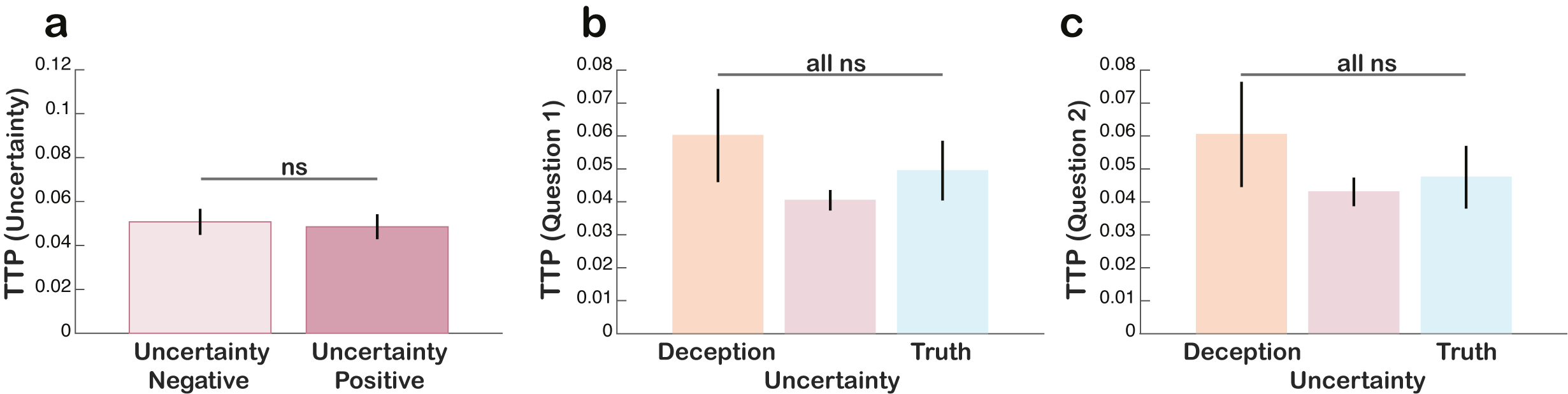


**Figure S2: Control Analyses. a) Trough-to-peak amplitude** for Negative Uncertainty and Positive Uncertainty conditions represented in light and dark pink respectively. Since they were not significantly different, they were merged in a single Uncertainty condition. **b) Trough-to-peak amplitude (Question 1)** for Deception, Uncertainty, and Truth conditions represented in orange, pink, and light blue respectively. Number of participants per condition: Deception = 24, Uncertainty = 31, Truth = 24**. c) Trough-to-peak amplitude (Question 2)** for Deception, Uncertainty, and Truth conditions represented in orange, pink, and light blue respectively. Number of participants per condition: Deception = 17, Uncertainty = 27, Truth = 24. Bars represent the mean value for each condition. Error bars represent ± 1 standard error. All comparisons are non-significant (ns).
